# Neglected Tropical Diseases and Sudden Cardiac Death: The NET-Heart Project

**DOI:** 10.31083/j.rcm2307254

**Published:** 2022-07-15

**Authors:** Andrés F. Miranda-Arboleda, Luis Gerardo González-Barrera, Kiera Liblik, Juan Farina, Ezequiel José Zaidel, Clara Saldarriaga, Zier Zhou, Reem Al-Rawi, José Patricio López-López, Jorge P. Juarez-Lloclla, Shyla Gupta, Dorairaj Prabhakaran, R Krishna Kumar, Álvaro Sosa-Liprandi, Adrian Baranchuk

**Affiliations:** ^1^Division of Cardiology, Kingston Health Science Centre, Queen’s University, Kingston, ON K7L 2V7, Canada; ^2^Cardiology Department, Pablo Tobón Uribe Hospital, 050034 Medellín, Colombia; ^3^Medical Society of 20 of November National Medical Center - Cardiology, 03229 Mexico City, Mexico; ^4^Department of Medicine, Translational Medicine, Queen’s University, Kingston, ON K7L 2V7, Canada; ^5^Department of Cardiovascular and Thoracic Surgery, Mayo Clinic, Phoenix, AZ 85054, USA; ^6^Cardiology Department, Sanatorio Güemes, and School of Medicine, University of Buenos Aires, C1180AAX Buenos Aires, Argentina; ^7^Cardiology Service, Clínica CardioVID, Universidad de Antioquia, 050034 Medellín, Colombia; ^8^Atherosclerosis, Genomics and Vascular Biology Division, University of Ottawa Heart Institute, Ottawa, ON K1Y 4W7, Canada; ^9^Department of Medicine, Queen's University, Kingston, ON K7L 2V7, Canada; ^10^Cardiology Unit, Hospital Universitario San Ignacio/Pontificia Universidad Javeriana, 110231 Bogotá, Colombia; ^11^Instituto Masira, Universidad de Santander, 680008 Bucaramanga, Colombia; ^12^Departament of Cardiology, Hospital de Apoyo II Santa Rosa, 20001 Piura, Peru; ^13^Faculty of Health Sciences, Queen’s University, Kingston, ON K7L 2V7, Canada; ^14^Centre for Chronic Conditions and Injuries, Public Health Foundation of India, 122002 Gurugram, India; ^15^London School of Hygiene and Tropical Medicine, WC1E 7HT London, UK; ^16^Amrita Institute of Medical Sciences and Research Centre, Cochin, 682041 Kerala, India

**Keywords:** neglected tropical diseases, sudden cardiac death, myocarditis, ventricular arrhythmias

## Abstract

Sudden cardiac death (SCD) is responsible for approximately 6% of global mortality and 25% of cardiovascular (CV) deaths. SCD has been traditionally linked to coronary artery disease, valvular heart disease, cardiomyopathies, and genetic arrhythmia disorders. However, advancements in care for these diseases have not translated to a proportional reduction in SCD. This suggests an important role of underrecognized contributing pathologies. Neglected tropical diseases (NTDs) are a group of illnesses prevalent in tropical and sub-tropical regions which have been understudied partially due to their high prevalence in marginalized populations. The relationship between SCD and Chagas disease has been well-established, though emerging literature suggests that other NTDs with CV involvement may lead to fatal arrhythmias. Additionally, specific therapies for a subset of NTDs put patients at increased risk of malignant arrhythmias and other cardiac complications. This review aims to summarize the association between a group of selected NTDs and SCD.

## 1. Introduction

**Fig. 1.**
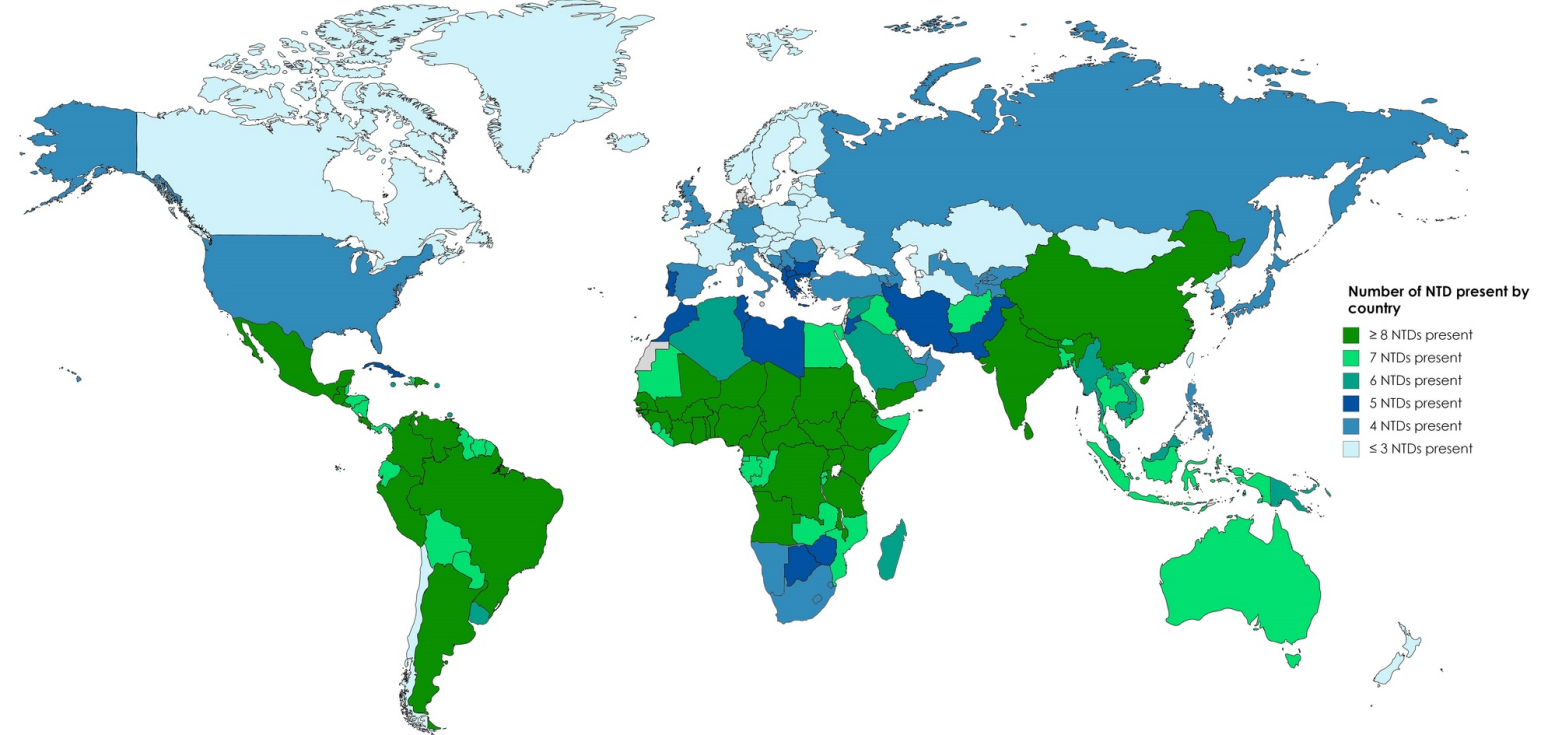
**Global burden of NTDs by country**. NTDs, Neglected tropical diseases. Data from World Health Organization Control of Tropical Neglected Disease. Modified from Burgos LM, *et al*. Glob Heart. 2020; 15: 60 [1].

Neglected tropical diseases (NTDs) predominantly impact tropical and sub-tropical regions of several low and low-middle-income countries (LMICs) (Fig. 1) [1]. Considering that the burden of traditional risk factors cannot fully explain the high rates of cardiovascular (CV) diseases in these regions, there is increasing recognition of the contribution of NTDs [2, 3].

The incidence of sudden cardiac death (SCD) is higher in LMICs. Thus, socioeconomic status seems to be inversely correlated with SCD [4, 5, 6], and has become a critical public health problem in these populations. It has been established that patients coming from LMICs have a higher burden of CV risk factors, CV disease burden, and mortality than those in high-income countries [3, 7]. However, the burden of traditional risk factors and limited access to healthcare is insufficient to explain this socioeconomic gradient of SCD [3, 7]. Further, there is apaucity of high-quality health data collected in these regions, making it more difficult to delineate the contributors to SCD [8].

There is an urgent need to accurately understand the burden of SCD in already underserved regions. This requires the development of an in-depth understanding of the contribution of NTDs. The present review summarizes the literature on the relationship between a selected group of NTDs and SCD.

## 2. Methods

A narrative review of the literature was performed. Databases searched included PubMed, Scielo, and Google Scholar. Databases were reviewed without language or publication date restriction. Reference lists, abstracts, policies, international society guidelines, and governmental healthcare organizations’ statements were also reviewed. The search was performed independently by each coauthor with the criteria of studies describing NTDs and their relationship with SCD. The selection of relevant NTDs to discuss was based on previous systematic reviews carried out as part of the Interamerican Society of Cardiology (SIAC) NET-Heart Project. The conditions identified as having a potential increased risk of SCD were included in this review [9, 10, 11, 12, 13, 14, 15, 16].

## 3. Specific NTDs Related to SCD

### 3.1 Chagas Disease

Chagas disease (CD) is a protozoan infection caused by *Trypanosoma cruzi* (*T. cruzi*). It is transmitted to mammals via insects belonging to the *Reduviidae* family (triatomine) [17]. CD is endemic to the southern United States and most countries in Central and South America. Although 65 million people live in endemic areas, most infected individuals are undiagnosed or have significant limitations accessing medical care [14, 18]. Due to migration and globalization, CD has become a global health concern with an increasing presence of chronic complications in the United States, Europe, and Asia [19, 20, 21].

The most common clinical implications of chronic CD are related to CV compromise. These impacting 30% to 40% of patients after the acute infection, persisting for up to three decades [17]. CV involvement of CD includes bradycardia, atrioventricular (AV) block, intraventricular conduction abnormalities, autonomic dysfunction, syncope, heart failure (HF), left ventricular (LV) dysfunction, structural abnormalities, and SCD [17].

Approximately 50,000 people die annually due to CD. SCD represents the most common cause of death in these patients (60%), followed by HF (25%), and thromboembolic events (15%) [22]. Premature death in patients with CD is of particular concern. It affects a high proportion of young people, which contributes to economic impact and social burden. SCD can be the first manifestation of CV involvement. Ventricular arrhythmias (VAs) are thought to be the principal cause of death in this subset of patients [23]. Other arrhythmic conditions leading to SCD in CD include advanced AV block and pulseless electrical activity [24, 25].

**Fig. 2.**
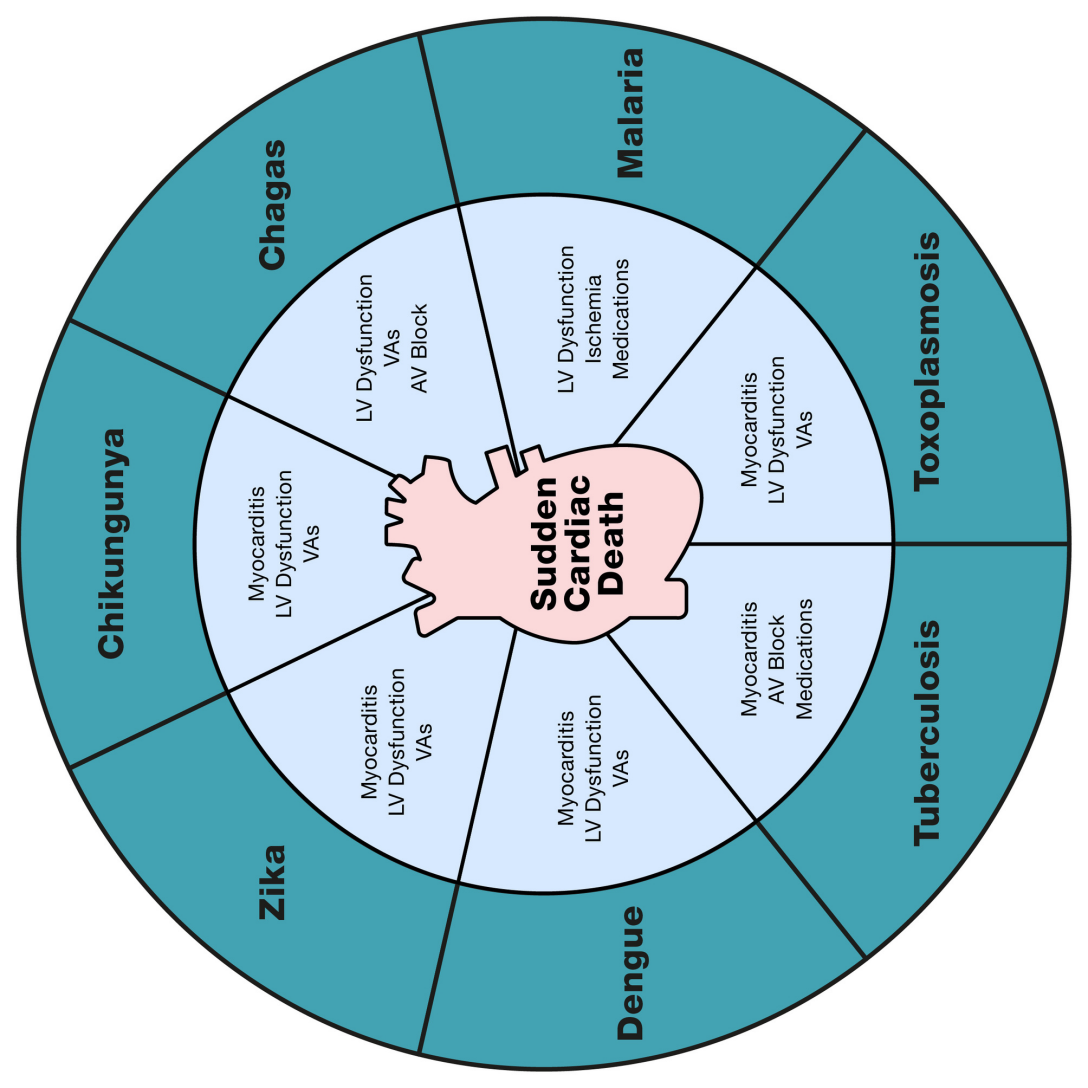
**NTDs and their pathophysiological mechanisms related to SCD**. AV, Atrioventricular; LV, Left ventricle; VAs, Ventricular arrhythmias.

The pathophysiology of SCD in CD is multifactorial. It includes myocardial inflammation, fibrosis, scarring, and remodeling caused by parasite invasion to the myocardium. Additionally, microvascular derangements, autonomic dysregulation, and autoimmunity may occur (Fig. 2) [26, 27]. Risk factors for the development of SCD in CD include male sex, previous VAs, LV dysfunction, syncope, bradycardia, late potentials on signal-averaged electrocardiography, and myocardial fibrosis [28]. Based on these factors, prediction scores have been developed to estimate the risk of SCD in this population (Table 1, Ref. [15]) [29, 30, 31, 32].

**Table 1.** **Scores to predict the risk of mortality in patients with Chagas disease**.

Rassi score (Prediction of mortality)		Ribeiro score (Prediction of mortality)		Sousa score (Prediction of SCD)	
**Variable**	**Points**	**Variable**	**Points**	**Variable**	**Points**
Cardiomegaly	5	LVEF <50%	1	QT dispersion	3
NYHA III or IV	5	Ventricular tachycardia (Holter or Stress test)	1	Syncope	2
LV wall motion abnormalities*	3	Prolonged QRS (>130 on ECG or >150 on signal‐averaged ECG)	1	LV function	1
Non-sustained ventricular tachycardia	3			Presence of PVC	1
Male sex	2				
Low QRS	2				
**Risk categories**	**Points**	**Risk categories**	**Points**	**Risk categories**	**Points**
Low	0–6	Low	0–1	Low	0–2
Intermediate	7–11	Intermediate	2	Intermediate	3–4
High	12–20	High	3	High	≥5
**5-year risk of death**	**%**	**5-year risk of death**	**%**	**5-year risk of SCD**	**%**
Low	2	Low	1	Low	1.5
Intermediate	18	Intermediate	20	Intermediate	25
High	61	High	50	High	51
**10-year risk of death**	**%**				
Low	10				
Intermediate	44				
High	88				

* Accounts for segmental or global wall motion abnormalities. ECG, Electrocardiogram; NYHA, New York Health Association; LV, Left ventricle; LVEF, Left ventricle ejection fraction; PVC, Premature ventricular contraction; SCD, Sudden cardiac death. Modified from reference [15].

Treatment of VAs related to CD remains an area of controversy. Antiarrhythmic drugs successfully reduce arrhythmic burden, but they do not reduce mortality [24]. Further, there is limited evidence on the utility of amiodarone for the treatment of VAs in CD [33].

Catheter ablation is an alternative for patients who have recurrent VAs despite antiarrhythmic therapy or who are not able to tolerate medications [34]. The arrhythmia substrate in CD is complex because it frequently includes multiple foci, large scars, and epicardial circuits which influence procedure outcomes [24]. Recently published studies suggest that the endo/epicardial approach for catheter ablation improves results [35]. When VT recurs after ablation, neuraxial modulation and surgical cardiac denervation have shown effectiveness in reducing arrhythmia burden [34, 36]. Access to complex electrophysiology care is severely restricted for populations living in LMICs. As a result, invasive treatment options may be limited in the most affected regions.

### 3.2 Malaria

Malaria is caused by the protozoa *Plasmodium (P.) falciparum*, *knowlesi*, *vivax*, and *ovale*. It is transmitted by infected female *Anopheles* mosquitoes [11]. Malaria is endemic to Asia and Oceania as well as South and Central America, with the highest risk of infection occurring in sub-Saharan Africa [11]. There is a considerable burden of disease associated with malaria, as it was responsible for approximately 627,000 deaths in 2020 [37].

The pathophysiology of CV consequences secondary to malaria is not well understood. It may involve a pro-inflammatory cytokine response, erythrocyte sequestration, and increased cytoadherence to endothelial linings [38, 39]. Additionally, the profound anemia produced by hemolysis and suppression of hematopoiesis in malaria may impair cardiac function. The proposed mechanism is the induction of cardiac stress leading to eccentric LV hypertrophy and associated volume overload, secondary tissue ischemia, and hypoxia. Sequestration of infected red blood cells in cardiac capillaries and subsequent blood flow obstruction may also cause LV dilation, ischemia, VAs, and SCD (Fig. 2) [11]. Patients with complicated malaria that progresses to severe sepsis or septic shock often have myocardial dysfunction, with ventricular dilation and reduced ejection fraction. This increasesthe risk of SCD [40].

Electrocardiographic changes have been reported in complicated malaria, such as ST-segment elevation secondary to ischemia and prolonged QTc intervals. These pathologies increase the risk of developing fatal VAs and SCD [40]. Notably, polymorphic ventricular tachycardia is a known life-threatening complication of antimalarial drugs that can lead to SCD [41, 42]. Tachycardia and tachyarrhythmias seen in malaria are also consequences of the anti-malarial drugs amodiaquine and halofantrine. Finally, quinine, another extensively used drug in treating severe malaria, is known to cause QT interval prolongation, *Torsades des pointes*, high degree AV block, and SCD [41].

Although there is no specific treatment to reduce the risk of SCD in malaria, the complicated forms of the infection can be treated with intravenous quinidine. Patients require continuous intensive care monitoring, including serial ECG in those treated with cardiotoxic antimalarials. Hence recognizing the CV complications of these drugs and their electrocardiographic manifestations facilitates the use of the ECG as a monitoring tool. Severe anemia and high levels of parasitemia can be managed with blood transfusions and supplemental oxygen to reduce the risk of arrhythmia, HF, and secondary myocardial injury [11].

### 3.3 Toxoplasmosis

Toxoplasmosis is caused by the parasite *Toxoplasma gondii* which spreads to humans through the consumption of undercooked meat, contaminated water, or contact with mammal feces [16]. Toxoplasmosis is typically mild and self-resolving in healthy hosts but may lead to severe disease in immunocompromised patients [16]. The CV involvement in toxoplasmosis typically manifests as myocarditis, where inflammatory cellular infiltrates occur with or without myocyte necrosis [43]. Though uncommon and often subclinical, toxoplasmic myocarditis is associated with progressive cardiac dysfunction (Fig. 2) [16]. To date, 12 cases of cardiac toxoplasmosis with SCD have been reported in the literature. All patients were immunocompromised, and 11 suffered from human immunodeficiency virus and acquired immunodeficiency syndrome. The remaining patient underwent a heart transplant [43, 44, 45, 46].

Reports of cardiac toxoplasmosis may be uncommon due to the requirement of invasive diagnostic strategies. Extensive tissue sampling of the heart might be needed to confirm the diagnosis as lesions often localize in the myocardium of the LV [43]. Thus, the connection between SCD and toxoplasmosis has not been extensively studied and requires further investigation.

Acquired toxoplasmosis in immunocompetent patients with mild symptoms does not require specific treatment. Though, intervention is necessary when severe systemic compromise occurs. CV toxoplasmosis has been successfully treated with a combination of pyrimethamine, sulfadiazine, and folinic acid [16].

Based on the currently available information, immunocompromised patients with toxoplasmic myocarditis are at a considerable risk of related SCD. Clinicians should take appropriate measures to prevent this fatal outcome. These may include managing symptoms and promptly treating CV complications such as arrhythmias and LV dysfunction. In highly endemic areas, providing prophylaxis for organ transplant recipients is recommended [47].

### 3.4 Tuberculosis 

Tuberculosis is the leading global cause of mortality due to infectious diseases and is among the top 10 leading causes of death worldwide [48]. Though it is predominantly a pulmonary disease, cardiac involvement is not infrequent in tuberculosis and contributes to morbidity and mortality [13].

CV involvement in tuberculosis occurs due to compromise of the pericardium, myocardium, and aorta. Pericardial disease is present in up to 5% of all cases of pulmonary disease and may manifest as acute pericarditis, pericardial effusion, or chronic constrictive pericarditis [13]. Myocarditis is rare, with an estimated incidence of less than 1% in these patients [49, 50]. *Mycobacterium tuberculosis* can spread to the myocardium in three ways: direct extension, retrograde spread via lymphatics, or via the hematogenous route [13, 50].

Once the myocardium is affected, CV involvement may progress to fulminant myocarditis, HF, conduction system disturbances, AV block, prolonged QT, and/or SCD [49, 51]. SCD in tuberculosis may be secondary to VAs induced by myocardial necrosis and damage of the interventricular septum, leading to electro-mechanical uncoupling. VT and ventricular fibrillation resulting in asystole may also occur (Fig. 2). Septal conduction system disturbances due to direct tuberculosis infiltration have also been proposed to cause death [52]. Likewise, HF leading to dilated cardiomyopathy can predispose patients to SCD [51, 53].

Typically, a four-drug conjugated regimen (isoniazid, rifampicin, ethambutol, and pyrazinamide) is the mainstay of anti-tuberculosis therapy [54]. Adverse CV events secondary to tuberculosis medications are rare. Notably, isoniazid treatment has been associated with QT interval prolongation and polymorphic ventricular tachycardia [54]. No link between other anti-tuberculosis medications and SCD has been reported.

There is insufficient data to guide a specific treatment for myocarditis in tuberculosis or prevent its complications [55]. Thus, prevention strategies for SCD should be prioritized. A useful and applicable algorithm has been published previously, suggesting that early diagnosis of cardiac tuberculosis, improved access to diagnostic tests, and combined antituberculosis medications with steroids are the basis for reducing associated mortality [13].

### 3.5 Dengue Virus 

Dengue virus (DENV) is a flavivirus transmitted by the vector *Aedes aegypti* which is predominantly found in tropical and subtropical regions [56]. Most patients infected with DENV are asymptomatic or have a mild illness. A minority of patients develop severe disease and subsequent CV complications [9]. Risk factors for CV involvement include age >65 years and CV comorbidities such as hypertension, diabetes mellitus, and ischemic heart disease [57].

CV complications of DENV are seen in up to 12.5% of patients [9]. Manifestations of CV compromise include bradyarrhythmias, tachyarrhythmias, myocarditis, LV dysfunction, and pericarditis [9, 57].

DENV infection may lead to three interrelated pathophysiological processes associated with SCD: LV systolic dysfunction, myocarditis, and arrhythmias (Fig. 2) [57]. These processes act as triggers and modulators of cardiac electrical instability. Arrhythmias can progress to VF (with or without previous VT) [58], and less frequently severe bradyarrhythmia with consequent SCD [59].

There is no specific treatment for DENV infection. In general, early identification of CV involvement and timely hemodynamic support should be initiated to treat patients with severe cardiac affection [9, 57, 59].

### 3.6 Zika Virus 

Zika virus (ZIKV) is an arbovirus that is part of the *Flaviviridae* family. It is mainly transferred to humans by *Aedes aegypti* and *Aedes Albopictus* mosquitoes [60]. It is also transmissible by asymptomatic hosts via sexual intercourse, blood transfusion, and transplacentally [61]. Currently, the ZIKV is mainly found in the Americas, Africa, the West Pacific, and Southeast Asia. Most patients with ZIKV disease do not present with any symptoms. Approximately 20% of patients with ZIKV have mild to moderate symptoms that usually resolve after two to seven days [15, 61].

CV involvement in ZIKV disease includes pericarditis, myocarditis, arrhythmias, and HF which may lead to SCD (Fig. 2). Specifically, the virus invades the cell through tyrosine-protein kinase 3 and ICAM-3 receptors, prompting pro-inflammatory cytokine release. Subsequently, inflammatory or autoimmune damage triggers cellular apoptosis [15]. Myocarditis caused by ZIKV may only present as nonspecific chest pain or no symptoms at all and is usually undiagnosed. Thus, myocardial damage may go unnoticed. This can develop into HF, myocardial infarction, cardiogenic shock, and even SCD during the acute stage of infection [15, 62, 63].

Early diagnosis and treatment of CV complications are the cornerstone to reduce the risk of sudden death in ZIKV disease patients. Acute symptoms, such as fever and fatigue may resolve with adequate hydration, rest, and antipyretics. However, severe infection may require intravenous polyvalent immunoglobulins [15].

### 3.7 Chikungunya Virus 

Chikungunya virus (CHIKV) is an enzootic ribonucleic acid virus that is transmitted by female *Aedes albopictus* and *Aedes aegypti* mosquitoes to humans [10]. CHIKV disease commonly presents in patients with acute fever and severe arthralgia. Systemic involvement, such as CV complications, is only seen in severe cases [10]. Patients who have comorbidities or are elderly are at a greater risk of systemic compromise [10].

Those with severe CHIKV are at elevated risk of developing myocarditis, arrhythmias, and HF (Fig. 2). CHIKV directly damages muscle fibers by puncturing myocytes, thereby augmenting inflammation, and producing secondary necrosis [10]. The most common CV complication related to CHIKV is myocarditis, which may contribute to further developing arrhythmias, myocardial necrosis, and SCD [10, 64, 65].

The early recognition and treatment of CHIKV are critical to preventing CV manifestations [10]. Myocarditis does not always present with symptoms before evolving to SCD [64]. Supportive measures such as cardiac monitoring, inotropic drugs, and oxygenation, can be used in severe cases [10]. Currently, there is no treatment or vaccine against CHIKV disease. However, there are certain proposed treatments for CHIKV cardiac complications. Notably, intravenous hydrocortisone may be administered to treat myocarditis. Although there is uncertainty regarding its benefit to mortality [10].

## 4. Discussion

Implementing preventative measures is the first step in reducing the burden of SCD in NTDs. Further, raising awareness will aid in early diagnosis and recognition of the CV involvement. Additionally, the pathophysiology of SCD in NTDs must be elucidated to improve evidence-based care [22, 51].

**Fig. 3.**
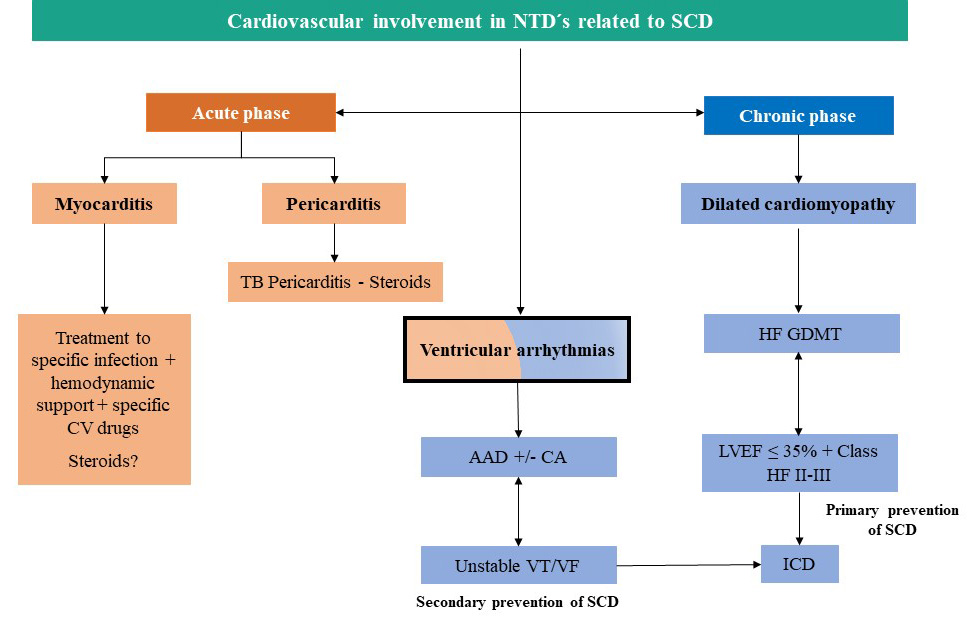
**General approach for specific therapies to prevent SCD in NTDs**. AAD, antiarrhythmic drugs; CA, Catheter ablation; CV, cardiovascular; ICD, implantable cardioverter defibrillator; GDMT, guideline-directed management and therapy; HF, heart failure; LVEF, left ventricular ejection fraction; TB, tuberculosis; VT, ventricular tachycardia; VF, ventricular fibrillation.

Myocarditis is the primary CV manifestation of most NTDs. It is frequently associated with the development of acute HF, arrhythmias, and SCD [9, 10, 11, 13, 15, 16, 17]. In general, the standard treatment for each NTD leads to the resolution of myocarditis [42, 66, 67]. In severe cases, steroid or mechanical circulatory support may be considered in addition to conventional therapy [42, 68, 69, 70, 71]. If HF develops, management is based on current HF guidelines (Fig. 3) [72, 73].

Risk stratification tools for SCD have been established for patients with chronic Chagas cardiomyopathy (Table 1) [30]. Similar tools have not been developed for other NTDs due to the limited number of cases reported in the literature and limited understanding of the mechanisms of SCD [2, 3, 12].

The indications for preventative Implantable Cardioverter Defibrillators (ICDs) in NTD patients have been extrapolated from international guidelines [74]. However, these recommendations are limited to patients with an LV ejection fraction less than 35% and New York Heart Association HF class II-III (Fig. 3) [72, 73]. In this setting, cardiac magnetic resonance (CMR) with late gadolinium enhancement could contribute to risk stratification. CMR is useful, as the presence of fibrosis is associated with an increased risk of SCD regardless of the ejection fraction [75].

Patients with CD cardiomyopathy have received specific attention in the literature [76]. Observational data suggests that in patients with CD with a LV ejection fraction less than 41% and life-threatening arrhythmias the treatment with a combination of an ICD and amiodarone reduces all-cause mortality by 72% and rates of SCD in 95% [77]. ICD is also indicated in the secondary prevention of SCD in patients with previous aborted cardiac arrest attributable to VAs (Fig. 3) [72, 73].

Antiarrhythmic drugs and catheter ablation can be considered ancillary interventions in patients with VAs after ICD implant to reduce the risk of appropriate and inappropriate shocks, which are associated with an increase in mortality and a significant reduction in quality of life (Fig. 3) [78, 79].

**Fig. 4.**
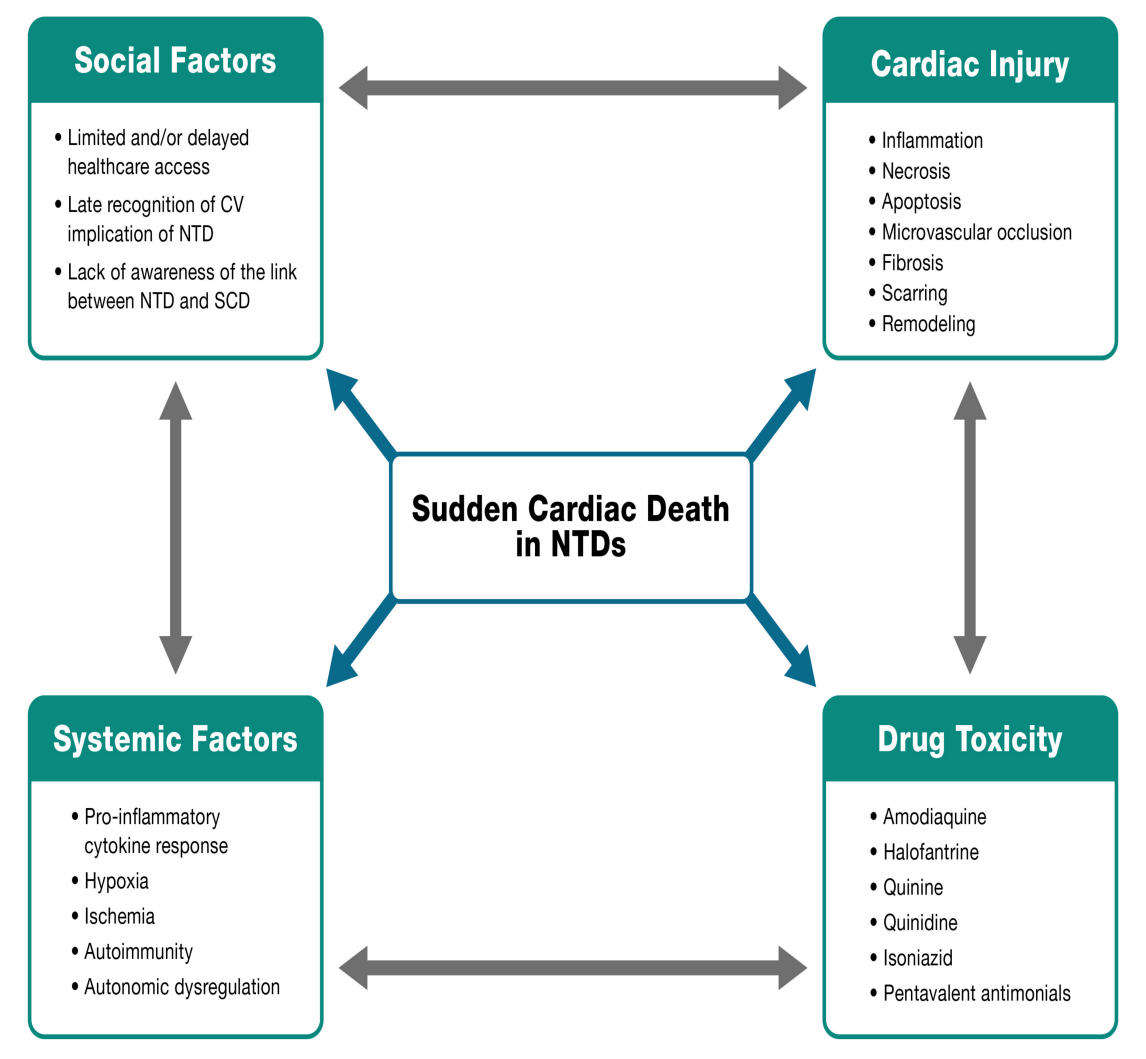
**NET Heart’s deadly quartet of SCD in NTDs**. CV, cardiovascular.

The relationship between NTDs and SCD is still poorly understood. To give a broad overview of this complex topic, we propose the *NET Heart’s deadly quartet of SCD in NTDs* (Fig. 4) which highlights four cornerstones:

(1) Social factors: Poor access to optimal and timely healthcare, late recognition of CV involvement in NTDs, and limited recognition of the link between NTDs and SCD.

(2) Systemic factors: Pro-inflammatory cytokine response, hypoxia, ischemia, autoimmunity, and autonomic dysregulation.

(3) Cardiac factors: Inflammation, necrosis, apoptosis, microvascular occlusion, fibrosis, scarring, and remodeling.

(4) Drugs: Some medications required for the treatment of NTDs have cardiotoxic effects that can be related to SCD such as quinine, quinidine, antimonials, and isoniazid.

These considerations will allow clinicians to recognize the interconnection between NTDs and SCD in regions with the highest prevalence. Thus, allowing to focus on developing multimodal approaches that reduce the impact generated by these unrecognized and neglected causes of SCD.

Populations living in poverty are at a higher risk of SCD associated with NTDs identifying several roadblocks to accessing optimal care. These considerations impact clinical decisions and require the development of approaches tailored to low-resource settings.

The main limitation of the present review is that it was not systematic. Additionally, it was based on a topic with limited associated literature. Further, NTDs encompass an extensive and heterogeneous group of diseases. Discussing in the proposed CV involvement of every NTD is beyond the scope of this document

## 5. Conclusions

The relationship between NTDs and SCD is complex, and the literature continues to be scarce on this topic. Some NTDs share pathophysiological mechanisms that can contribute to the development of SCD. However, multiple factors trigger and perpetuate the risk of death in this heterogeneous group of patients. We propose a holistic view of this public health problem entitled the *NET Heart’s deadly quartet of SCD in NTDs* aiming to facilitate the understanding and the implementation of strategies to reduce the impact generated by these conditions.

## References

[1] Burgos LM, Farina J, Liendro MC, Saldarriaga C, Liprandi AS, Wyss F (2020). Neglected Tropical Diseases and Other Infectious Diseases Affecting the Heart. The NET-Heart Project: Rationale and Design. *Global Heart*.

[2] Saldarriaga C, Baranchuk A (2022). *Neglected Tropical Diseases and other Infectious Diseases affecting the Heart. 1st edn.*.

[3] Moolani Y, Bukhman G, Hotez PJ (2012). Neglected tropical diseases as hidden causes of cardiovascular disease. *PLoS Neglected Tropical Diseases*.

[4] Reinier K, Thomas E, Andrusiek DL, Aufderheide TP, Brooks SC, Callaway CW (2011). Socioeconomic status and incidence of sudden cardiac arrest. *Canadian Medical Association Journal*.

[5] Medhekar AN, Adhikari S, Abdul‐Al AS, Matinrazm S, Kancharla K, Bhonsale A (2019). Lower socioeconomic status is associated with increased long-term mortality after sudden cardiac arrest. *Clinical Cardiology*.

[6] Sliwa K, Wilkinson D, Hansen C, Ntyintyane L, Tibazarwa K, Becker A (2008). Spectrum of heart disease and risk factors in a black urban population in South Africa (the Heart of Soweto Study): a cohort study. *The Lancet*.

[7] Timmis A, Townsend N, Gale CP, Torbica A, Lettino M, Petersen SE (2020). European Society of Cardiology: Cardiovascular Disease Statistics 2019. *European Heart Journal*.

[8] Vedanthan R, Fuster V, Fischer A (2012). Sudden cardiac death in low- and middle-income countries. *Global Heart*.

[9] Araiza-Garaygordobil D, García-Martínez CE, Burgos LM, Saldarriaga C, Liblik K, Mendoza I (2021). Dengue and the heart. *Cardiovascular Journal of Africa*.

[10] Cotella JI, Sauce AL, Saldarriaga CI, Perez GE, Farina JM, Wyss F (2021). Chikungunya and the Heart. *Cardiology*.

[11] Gupta S, Gazendam N, Farina JM, Saldarriaga C, Mendoza I, López-Santi R (2021). Malaria and the Heart: JACC State-of-the-Art Review. *Journal of the American College of Cardiology*.

[12] Iomini PA, Parodi JB, Farina JM, Saldarriaga C, Liblik K, Mendoza I (2021). Neglected tropical diseases and their impact on cardiovascular health (The NET-Heart Project). *Medicina (B Aires)*.

[13] López‐López JP, Posada‐Martínez EL, Saldarriaga C, Wyss F, Ponte‐Negretti CI, Alexander B (2021). Tuberculosis and the Heart. *Journal of the American Heart Association*.

[14] Miranda-Arboleda AF, Zaidel EJ, Marcus R, Pinazo MJ, Echeverria LE, Saldarriaga C (2021). Roadblocks in Chagas disease care in endemic and nonendemic countries: Argentina, Colombia, Spain, and the United States. The NET-Heart project. *PLoS Neglected Tropical Diseases*.

[15] Scatularo CE, Ballesteros OA, Saldarriaga C, Mendoza I, Wyss F, Liprandi AS (2022). Zika & heart: A systematic review. *Trends in Cardiovascular Medicine*.

[16] Zhou Z, Ortiz Lopez HIA, Pérez GE, Burgos LM, Farina JM, Saldarriaga C (2021). Toxoplasmosis and the Heart. *Current Problems in Cardiology*.

[17] Miranda-Arboleda AF, Krishnan D, Zaidel EJ, Echeverría LE, Saldarriaga C, Farina JM, Saldarriaga C, Baranchuk A (2022). Chapter 5 - Cardiovascular Complications of Chagas’ Disease. *Neglected Tropical Diseases and other Infectious Diseases affecting the Heart. 1st edn.*.

[18] Saraiva RM, Mediano MFF, Mendes FS, Sperandio da Silva GM, Veloso HH, Sangenis LHC (2021). Chagas heart disease: an overview of diagnosis, manifestations, treatment, and care. *World Journal of Cardiology*.

[19] Velasco A, Morillo CA (2020). Chagas heart disease: a contemporary review. *Journal of Nuclear Cardiology*.

[20] Echeverria LE, Marcus R, Novick G, Sosa-Estani S, Ralston K, Zaidel EJ (2020). WHF IASC Roadmap on Chagas Disease. *Global Heart*.

[21] Miranda-Schaeubinger M, Chakravarti I, Freitas Lidani KC, Omidian Z, Gilman RH (2019). Systematic Review of the Epidemiology of Chagas Disease in the Americas: a Call for Standardized Reporting of Chagas Disease Prevalence. *Current Tropical Medicine Reports*.

[22] Keegan R, Yeung C, Baranchuk A (2020). Sudden Cardiac Death Risk Stratification and Prevention in Chagas Disease: A Non-systematic Review of the Literature. *Arrhythmia & Electrophysiology Review*.

[23] Lee BY, Bacon KM, Bottazzi ME, Hotez PJ (2013). Global economic burden of Chagas disease: a computational simulation model. *The Lancet Infectious Diseases*.

[24] Healy C, Viles-Gonzalez JF, Sáenz LC, Soto M, Ramírez JD, d’Avila A (2015). Arrhythmias in Chagasic Cardiomyopathy. *Cardiac Electrophysiology Clinics*.

[25] Bestetti RB, Cardinalli-Neto A (2008). Sudden cardiac death in Chagas’ heart disease in the contemporary era. *International Journal of Cardiology*.

[26] Machado FS, Tanowitz HB, Ribeiro AL (2013). Pathogenesis of Chagas Cardiomyopathy: Role of Inflammation and Oxidative Stress. *Journal of the American Heart Association*.

[27] Marin-Neto JA, Simoes MV, Rassi Junior A (2013). Pathogenesis of chronic Chagas cardiomyopathy: the role of coronary microvascular derangements. *Revista da Sociedade Brasileira de Medicina Tropical*.

[28] Di Toro D, Baranchuk A (2016). Sudden cardiac death in Chagas disease. *The International Cardiovascular Forum*.

[29] de Souza ACJ, Salles G, Hasslocher-Moreno AM, de Sousa AS, Alvarenga Americano do Brasil PE, Saraiva RM (2015). Development of a risk score to predict sudden death in patients with Chaga’s heart disease. *International Journal of Cardiology*.

[30] Rassi A, Rassi A, Little WC, Xavier SS, Rassi SG, Rassi AG (2006). Development and Validation of a Risk Score for Predicting Death in Chagas’ Heart Disease. *New England Journal of Medicine*.

[31] Ribeiro AL, Cavalvanti PS, Lombardi F, Nunes Mdo C, Barros MV, Rocha MO (2008). Prognostic value of signal-averaged electrocardiogram in Chagas disease. *Journal of Cardiovascular Electrophysiology*.

[32] Viotti R, Vigliano C, Lococo B, Petti M, Bertocchi G, Álvarez MG (2005). Indicadores clínicos de progresión de la miocarditis chagásica crónica. *Revista EspañOla De Cardiología*.

[33] Leite LR, Fenelon G, Simoes A, Silva GG, Friedman PA, Paola AAVD (2003). Clinical Usefulness of Electrophysiologic Testing in Patients with Ventricular Tachycardia and Chronic Chagasic Cardiomyopathy Treated with Amiodarone or Sotalol. *Journal of Cardiovascular Electrophysiology*.

[34] Romero J, Velasco A, Pisani CF, Alviz I, Briceno D, Díaz JC (2021). Advanced Therapies for Ventricular Arrhythmias in Patients with Chagasic Cardiomyopathy: JACC State-of-the-Art Review. *Journal of the American College of Cardiology*.

[35] Pisani CF, Romero J, Lara S, Hardy C, Chokr M, Sacilotto L (2020). Efficacy and safety of combined endocardial/epicardial catheter ablation for ventricular tachycardia in Chagas disease: a randomized controlled study. *Heart Rhythm*.

[36] Saenz LC, Corrales FM, Bautista W, Traina M, Meymandi S, Rodriguez DA (2016). Cardiac sympathetic denervation for intractable ventricular arrhythmias in Chagas disease. *Heart Rhythm*.

[37] World Health Organization (2021). Malaria key facts 2021. https://www.who.int/news-room/fact-sheets/detail/malaria.

[38] Costenaro P, Benedetti P, Facchin C, Mengoli C, Pellizzer G (2011). Fatal Myocarditis in Course of Plasmodium falciparum Infection: Case Report and Review of Cardiac Complications in Malaria. *Case Reports in Medicine*.

[39] Day NP, Hien T, Schollaardt T, Loc P, Chuong L, Chau T (1999). The Prognostic and Pathophysiologic Role of Pro- and Antiinflammatory Cytokines in Severe Malaria. *The Journal of Infectious Diseases*.

[40] Sadoh W, Uduebor J (2017). Electrocardiographic changes and troponin T levels in children with severe malaria anemia and heart failure. *Nigerian Journal of Clinical Practice*.

[41] Haeusler IL, Chan XHS, Guérin PJ, White NJ (2018). The arrhythmogenic cardiotoxicity of the quinoline and structurally related antimalarial drugs: a systematic review. *BMC Medicine*.

[42] White NJ (2007). Cardiotoxicity of antimalarial drugs. *The Lancet Infectious Diseases*.

[43] Lanjewar DN, Agale SV, Chitale AR, Joshi SR (2006). Sudden death due to cardiac toxoplasmosis. *The Journal of the Association of Physicians of India*.

[44] Bal A, Dhooria S, Agarwal R, Garg M, Das A (2014). Multiple and atypical opportunistic infections in a HIV patient with Toxoplasma myocarditis. *Cardiovascular Pathology*.

[45] Chimenti C, Del Nonno F, Topino S, Abbate I, Licci S, Grazia Paglia M (2007). Fatal myocardial co-infection by Toxoplasma gondii and Parvovirus B19 in an HIV patient. *AIDS*.

[46] Holliman RE, Johnson J, Burke M, Adams S, Pepper JR (1990). False-negative dye-test findings in a case of fatal toxoplasmosis associated with cardiac transplantation. *Journal of Infection*.

[47] Derouin F, Pelloux H (2008). Prevention of toxoplasmosis in transplant patients. *Clinical Microbiology and Infection*.

[48] World Health Organization (2019). Global Tuberculosis Report 2019. https://www.who.int/tb/publications/global_report/tb19_Exec_Sum_12Nov2019.pdf?ua=1.

[49] Behr G, Palin HC, Temperly JM (1977). Myocardial tuberculosis. *BMJ*.

[50] Rose AG (1987). Cardiac tuberculosis. A study of 19 patients. *Archives of Pathology & Laboratory Medicine*.

[51] Liu A, Hu Y, Coates A (2012). Sudden cardiac death and tuberculosis – how much do we know. *Tuberculosis*.

[52] Wallis PJ, Branfoot AC, Emerson PA (1984). Sudden death due to myocardial tuberculosis. *Thorax*.

[53] Bali HK, Wahi S, Sharma BK, Anand IS, Datta BN, Wahi PL (1990). Myocardial tuberculosis presenting as restrictive cardiomyopathy. *American Heart Journal*.

[54] Makarov LM, Chuprova SN, Garipov R, Sorokina EV, Poliakova EB, Kalinin LA (2003). Prolongation of the Q-T interval while taking isoniazid. *Terapevticheskii Arkhiv*.

[55] Dulin M, Pasi N, Benali K, Ducrocq G, Roriz M, Pellenc Q (2021). Management of patients with myocardial tuberculosis: a case series. *International Journal of Cardiology*.

[56] Guzman MG, Gubler DJ, Izquierdo A, Martinez E, Halstead SB (2016). Dengue infection. *Nature Reviews Disease Primers*.

[57] Yacoub S, Wertheim H, Simmons CP, Screaton G, Wills B (2014). Cardiovascular manifestations of the emerging dengue pandemic. *Nature Reviews Cardiology*.

[58] Lee JC, Cia CT, Lee NY, Ko NY, Chen PL, Ko WC (2022). Causes of death among dengue patients causes of death among hospitalized adults with dengue fever in Tainan, 2015: Emphasis on cardiac events and bacterial infections. *Journal of Microbiology, Immunology and Infection*.

[59] Parchani A, Krishnan VsG, Kumar VKS (2021). Electrocardiographic Changes in Dengue Fever: A Review of Literature. *International Journal of General Medicine*.

[60] Sharma V, Sharma M, Dhull D, Sharma Y, Kaushik S, Kaushik S (2020). Zika virus: an emerging challenge to public health worldwide. *Canadian Journal of Microbiology*.

[61] World Health Organization (2018). Zika virus key facts 2018. https://www.who.int/news-room/fact-sheets/detail/zika-virus.

[62] Cardona-Ospina JA, Henao-SanMartin V, Acevedo-Mendoza WF, Nasner-Posso KM, Martínez-Pulgarín DF, Restrepo-López A (2019). Fatal Zika virus infection in the Americas: a systematic review. *International Journal of Infectious Diseases*.

[63] Rajahram GS, Hale G, Bhatnagar J, Hiu J, Thayan R, William T (2019). Postmortem evidence of disseminated Zika virus infection in an adult patient. *International Journal of Infectious Diseases*.

[64] Traverse EM, Hopkins HK, Vaidhyanathan V, Barr KL (2021). Cardiomyopathy and Death Following Chikungunya Infection: An Increasingly Common Outcome. *Tropical Medicine and Infectious Disease*.

[65] Gonzalez Carta KA, Mendoza_Britto IJ, Finizola V, Morr I, Torres J, Meza Y (2016). Bradycardia as a Manifestation of Chikungunya Myocarditis. A New Threat to America. *Circulation*.

[66] Kirchhoff LV (2004). Parasitic diseases of the heart. *Frontiers in Bioscience*.

[67] Licht J, Diefenbach C, Stang A, Hartmann V, Bolte J, Kirsten D (2009). Tuberculoma of the myocardium: a rare case of intra-vitam diagnosis. *Clinical Research in Cardiology*.

[68] Aletti M, Lecoules S, Kanczuga V, Soler C, Maquart M, Simon F (2017). Transient myocarditis associated with acute Zika virus infection. *Clinical Infectious Diseases*.

[69] Hadem J, Schröder F, Winkler T, Gohrbandt B, Fischer D, Korte T (2006). One day from dyspnea to death–unsuccessful application of extracorporeal membrane oxygenation in toxoplasma myocarditis following bone marrow transplantation. *Clinical Research in Cardiology*.

[70] Minhas AM, Nayab A, Iyer S, Narmeen M, Fatima K, Khan MS (2017). Association of Zika Virus with Myocarditis, Heart Failure, and Arrhythmias: a Literature Review. *Cureus*.

[71] Montoya JG, Jordan R, Lingamneni S, Berry GJ, Remington JS (1997). Toxoplasmic Myocarditis and Polymyositis in Patients with Acute Acquired Toxoplasmosis Diagnosed during Life. *Clinical Infectious Diseases*.

[72] Al-Khatib SM, Stevenson WG, Ackerman MJ, Bryant WJ, Callans DJ, Curtis AB (2018). 2017 AHA/ACC/HRS Guideline for Management of Patients With Ventricular Arrhythmias and the Prevention of Sudden Cardiac Death: A Report of the American College of Cardiology/American Heart Association Task Force on Clinical Practice Guidelines and the Heart Rhythm Society. *Circulation*.

[73] Priori SG, Blomstrom-Lundqvist C, Mazzanti A, Blom N, Borggrefe M, Camm J (2015). 2015 ESC Guidelines for the management of patients with ventricular arrhythmias and the prevention of sudden cardiac death: The Task Force for the Management of Patients with Ventricular Arrhythmias and the Prevention of Sudden Cardiac Death of the European Society of Cardiology (ESC). Endorsed by: Association for European Paediatric and Congenital Cardiology (AEPC). *European Heart Journal*.

[74] Nunes MCP, Beaton A, Acquatella H, Bern C, Bolger AF, Echeverría LE (2018). Chagas Cardiomyopathy: an Update of Current Clinical Knowledge and Management: a Scientific Statement from the American Heart Association. *Circulation*.

[75] Cheong BYC, Muthupillai R, Wilson JM, Sung A, Huber S, Amin S (2009). Prognostic Significance of Delayed-Enhancement Magnetic Resonance Imaging: survival of 857 patients with and without left ventricular dysfunction. *Circulation*.

[76] Toro DD, Muratore C, Aguinaga L, Batista L, Malan A, Greco O (2011). Predictors of all-Cause 1-Year Mortality in Implantable Cardioverter Defibrillator Patients with Chronic Chagas’ Cardiomyopathy. *Pacing and Clinical Electrophysiology*.

[77] Gali WL, Sarabanda AV, Baggio JM, Ferreira LG, Gomes GG, Marin-Neto JA (2014). Implantable cardioverter-defibrillators for treatment of sustained ventricular arrhythmias in patients with Chagas’ heart disease: comparison with a control group treated with amiodarone alone. *Europace*.

[78] Poole JE, Johnson GW, Hellkamp AS, Anderson J, Callans DJ, Raitt MH (2008). Prognostic Importance of Defibrillator Shocks in Patients with Heart Failure. *New England Journal of Medicine*.

[79] Schron EB, Exner DV, Yao Q, Jenkins LS, Steinberg JS, Cook JR (2002). Quality of life in the antiarrhythmics versus implantable defibrillators trial: impact of therapy and influence of adverse symptoms and defibrillator shocks. *Circulation*.

